# Extracellular vesicles promote activation of pro-inflammatory cancer-associated fibroblasts in oral cancer

**DOI:** 10.3389/fcell.2023.1240159

**Published:** 2023-09-07

**Authors:** Julia Arebro, Rebecca Towle, Che-Min Lee, Kevin L. Bennewith, Cathie Garnis

**Affiliations:** ^1^ Department of Integrative Oncology, British Columbia Cancer Research Center, Vancouver, BC, Canada; ^2^ Division of ENT Diseases, Department of Clinical Science, Intervention and Technology, Karolinska Institutet, Stockholm, Sweden; ^3^ Department of ENT Diseases, Karolinska University Hospital, Stockholm, Sweden; ^4^ Interdisciplinary Oncology Program, University of British Columbia, Vancouver, BC, Canada; ^5^ Pathology and Laboratory Medicine, University of British Columbia, Vancouver, BC, Canada; ^6^ Division of Otolaryngology, Department of Surgery, University of British Columbia, Vancouver, BC, Canada

**Keywords:** cancer-associated fibroblast, tumor microenvironment, oral squamous cell carcinoma, extracellular vesicles, inflammation, tumorigenesis

## Abstract

**Introduction:** Oral squamous cell carcinoma (OSCC) is the most common form of head and neck cancer and has a survival rate of ∼50% over 5 years. New treatment strategies are sorely needed to improve survival rates—and a better understanding of the mechanisms underlying tumorigenesis is needed to develop these strategies. The role of the tumor microenvironment (TME) has increasingly been identified as crucial in tumor progression and metastasis. One of the main constituents of the TME, cancer-associated fibroblasts (CAFs), plays a key role in influencing the biological behavior of tumors. Multiple mechanisms contribute to CAF activation, such as TGFβ signaling, but the role of extracellular vesicles (EVs) in CAF activation in OSCC is poorly understood. Assessing the impact of oral cancer-derived EVs on CAF activation will help to better illuminate OSCC pathophysiology and may drive development of novel treatments options.

**Methods:** EVs were isolated from OSCC cell lines (Cal 27, SCC-9, SCC-25) using differential centrifugation. Nanoparticle tracking analysis was used for EV characterization, and Western blot to confirm the presence of EV protein markers. Oral fibroblasts were co-cultured with enriched EVs, TGFβ, or PBS over 72 h to assess activation. Flow cytometry was used to evaluate CAF markers. RNA collected from fibroblasts was extracted and the transcriptome was sequenced. Conditioned media from the co-cultures was evaluated with cytokine array profiling.

**Results:** OSCC-derived EVs can activate oral fibroblasts into CAFs that are different from those activated by TGFβ, suggesting different mechanisms of activation and different functional properties. Gene set enrichment analysis showed several upregulated inflammatory pathways in those CAFs exposed to OSCC-derived EVs. Marker genes for inflammatory CAF subtypes were also upregulated, but not in CAFs activated by TGFβ. Finally, cytokine array analysis on secreted proteins revealed elevated levels of several pro-inflammatory cytokines from EV-activated CAFs, for instance IL-8 and CXCL5.

**Discussion:** Our results reveal the ability of OSCC-derived EVs to activate fibroblasts into CAFs. These CAFs seem to have unique properties, differing from TGFβ-activated CAFs. Gaining an understanding of the interplay between EVs and stromal cells such as CAFs could lead to further insights into OSCC tumorigenesis and potential novel therapeutics.

## 1 Introduction

Cancer represents one of the leading causes of mortality and morbidity among humans. Oral squamous cell carcinoma (OSCC) affects mainly the floor of the mouth and the tongue, and has a 5-year survival rate of only 50%. Frequent late-stage diagnoses and high rates of recurrence are key drivers of this poor prognosis. There remains a clear need to develop new approaches for treating this disease. A better understanding of the molecular basis of OSCC progression and metastasis is needed to provide new disease management options and treatments strategies.

Tumor progression is no longer recognized as relating only to genetic mutation and uncontrollable growth in cancer cells. Different types of growth factors and cytokines secreted by tumor cells and stromal cells within a tumor, as well as signaling pathways induced by cell-cell interactions, are all thought to play key roles in tumorigenesis and metastasis (Meurette and Mehlen, 2018; Vitale et al., 2019). The tumor microenvironment (TME) is a dynamic space composed of cellular components, including fibroblasts, immune cells, inflammatory cells, epithelial cells, endothelial cells, and the extracellular matrix (ECM). The TME, ECM, and cellular components can regulate and influence each other (Zhou and Lu, 2017; Meurette and Mehlen, 2018; Roswall et al., 2018; Vitale et al., 2019; Vuong et al., 2019; Osman et al., 2020). Specifically, cancer-associated fibroblasts (CAFs) are one of the main cell types in the TME and are known to play a key role in influencing the biological behavior of tumors (Shoucair et al., 2020). Hence, CAFs have recently become of great interest in targeted medicine and tumor immunotherapy.

CAFs are a group of activated fibroblasts with a mesenchymal cell lineage and heterogeneity in the TME. They secrete a variety of active factors to participate in the generation and maintenance of cancer cell stemness, immune regulation, angiogenesis, metabolic response, ECM remodeling, internal environment stability, therapeutic resistance, and other biological processes (Shoucair et al., 2020). Fibroblasts can be activated and differentiate into a CAF phenotype through growth factors (TGFβ, HGF, FGF, PDGF), transcription factors (NF-κB and HSF-1), metalloproteinases, signal proteins, reactive oxygen species secreted by tumor cells or immune cells and by extracellular vesicles released by tumor cells (Dai et al., 2018; Ringuette Goulet et al., 2018; Ping et al., 2021). EVs are small membrane-encapsuled structures released by cells and serve as key mediators in cell-cell communication. Stromal cells, particularly fibroblasts, take up large numbers of cancer cell-derived EVs when compared to epithelial cells, making them important targets of EV-mediated cross-talk (Lee et al., 2016). CAF activation through cancer cell-derived EVs has however scarcely been shown in oral cancer (Ding et al., 2018; Jiang et al., 2019). We demonstrate the ability of OSCC-derived EVs to shape fibroblasts into CAFs that are different from canonical TGFβ-activated CAFs. CAFs activated by OSCC tumor-derived EVs have a distinct expression and secretome profile as compared to both non-activated fibroblasts or those activated by TGFβ, a finding that is suggestive of a unique function for these activated CAFs.

## 2 Materials and methods

### 2.1 Cell lines and cell culture

The oral cancer cell lines Cal 27 (ATCC^®^ CRL 2095™), SCC-9 (ATCC^®^ CRL1629™), and SCC-25 (ATCC^®^ CRL1628™) were obtained from the American Type Culture Collection (ATCC). Cal 27 cells were cultured in DMEM media supplemented with 10% FBS. SCC-9 and SCC-25 cells were cultured in DMEM/F12 media supplemented with 10% FBS and 400 ng/mL hydrocortisone. Cancer cell lines were used for no more than 2 months after thawing to prevent genetic drift. The human oral fibroblast line (HOrF #2640) was obtained from ScienCell™ Research Laboratories. Cells were cultured on coated surfaces using poly-L-lysine (ScienCell™ Research Laboratories Cat #0413) and in complete Fibroblast Medium (ScienCell™ Research Laboratories Cat #2301) containing 2% FBS. The HOrFs were used for no more than passage ten. All cells were cultured in a 37°C, 5% CO_2_ incubator.

### 2.2 EV isolation

Cancer cell lines (Cal 27, SCC-9, and SCC-25) were seeded into 15 cm plates. Forty-eight hours before reaching 90% confluency, normal media was replaced after PBS wash with media containing 1% FBS without noticeably affecting growth. The FBS was depleted of EVs by ultracentrifugation at 110,000 g for 16 h in order to reduce contamination from bovine EVs. Cells were allowed to excrete EVs into the media for 48 h, after which the conditioned media was collected and subjected to multiple rounds of centrifugation as previously described (Théry et al., 2018). Dead cells and cellular fragments were removed from media with 4°C centrifugation at 300 *g* for 10 min, 2,000 g for 20 min, and 10,000 g for 30 min, with the precipitate being discarded at each interval. EVs were precipitated using an ultracentrifuge at 110,000 g at 4°C for 90 min, after which the supernatant was removed, and the pellet was rinsed with PBS. EVs were re-precipitated with an additional 110,000 g spin for 90 min. NanoSight (Malvern, United Kingdom) nanoparticle tracking analysis was assessed to determine EV particle data. Vesicle flow was analyzed in a diluted sample for optimal particle range for the machine, and each sample was analyzed in triplicate for 60 s.

### 2.3 Fibroblasts co-cultured with EVs

HOrFs were seeded in six well plates with 2 mL of media. EVs, PBS, or TGFβ was then added three times with 24 h intervals in between (total time of co-culture: 72 h). The amount of EVs added at each timepoint was determined after titration assays evaluated with microscopy (to determine cell death) and flow cytometry (using the same panel of CAF markers as described below to assess CAF activation). In detail, when HOrFs had reached 30% confluency, filtered EVs isolated from oral cancer cell lines and resuspended in PBS (25 µL per well) were added to the culture media (22.6 × 10^9^ EV particles per well) while 25 µL of PBS was added per well to the PBS controls and 20 ng/well of TGF-beta 1 protein with carrier (R&D systems, final concentration 10 ng/μL after reconstitution) resuspended in 25 µL PBS was added per well to the TGFβ controls. After an additional 24 h and 48 h, the media was replaced with 2 mL/well of EV supplemented fresh media (45.2 × 10^9^ EV particles per well) for samples co-cultured with EVs, 2 mL/well of PBS-supplemented fresh media to the PBS controls, and 20 ng/well TGFβ (final concentration 10 ng/mL) resuspended in 25 µL PBS supplemented fresh media (2 mL/well) for the TGFβ controls.

### 2.4 Flow cytometry

HOrFs were lifted 24 h after the third treatment of EVs or TGFβ had been added, using EDTA 0.04%. Cells were stained with the following monoclonal antibodies: anti-alpha-Smooth Muscle Actin (αSMA) (Novus Biologicals Cat #NBP2-34760APC), anti- Human Fibroblast Activation Protein alpha (FAP) (R&D Systems Cat #FAB3715P-100), anti-Podoplanin (PDPN) (BioLegend Cat #337006), anti-CD29 (BioLegend Cat #303020), and anti-PDGFRβ (Cell Signaling Technology Cat #3169S) along with Brilliant Violet 421™ Donkey anti-rabbit IgG (BioLegend Cat #406410). All antibodies were titrated for optimum concentration before use, with dilutions ranging from 1:50 to 1:400. eBioscience™ Foxp3/Transcription Factor Staining Buffer Set (Invitrogen) was used for staining intracellular protein. Cells were identified based on forward and side scatter properties and singlets. Dead cells were excluded using Fixable Viability Dye eFluor (ThermoFisher Cat #65-0865-14). Gates on antibodies were performed using fluorescence minus one (FMO) controls. Cells were analyzed on an LRSFortessa analyser (BD Biosciences), and data were processed using FlowJo software (Tree Star, Inc., Ashland, United States). EV co-cultures for each of the oral SCC cell lines were performed on different days, each with their own controls (PBS and TGFβ), hence we observe some variability among the various control samples when analyzed with flow cytometry.

### 2.5 RNA extraction from HOrFs and RNA sequencing

RNA from HOrFs co-cultured with EVs, TGFβ, or PBS (three replicates per group) was extracted in TRIzol per manufacturer instructions, 24 h after the third time EVs had been added. RNA expression profiling was performed at the Biomedical Research Centre at the University of British Columbia with 7,000-10,000 ng of total RNA as input. Sample quality control was performed using the Agilent 2,100 Bioanalyzer yielding RNA integrity number (RIN) 10 for all samples. Qualifying samples were then prepped following the standard protocol for the NEBnext Ultra ii Stranded mRNA (New England Biolabs). Sequencing was performed on the Illumina NextSeq2000 with Paired End 61bp × 61bp reads. Sequencing data was de-multiplexed using Illumina’s bcl2fastq2. De-multiplexed read sequences were then aligned to the *Homo sapiens*/Mus musculous (PAR-masked)/hg19 or mm10) reference sequence using STAR aligner (https://www.ncbi.nlm.nih.gov/pubmed/23104886).

### 2.6 Data analysis of RNA sequencing

Sequencing analysis resulted in an average of 26 million reads per sample. Assembly and differential expression were estimated using Cufflinks (http://cole-trapnell-lab.github.io/cufflinks/) through bioinformatics apps available on Illumina Sequence Hub, using FPKM values and comparing treatment group (EV or TGFβ treatment) versus control (PBS treatment). Corrected *p*-value <0.05 was considered significant. Pre-ranked Gene set enrichment analysis (GSEA) was performed on the counts expressed genes in each treatment group, using chip platform Human Gene Symbol with Remapping and Hallmarks and Biocarta gene sets from MSigDB (Liberzon et al., 2011). Enrichment map visualization in GSEA was used together with Cytoscape 3.9.1. Expression-based heat map of signature marker genes for CAF subtypes was performed according to instructions (Babicki et al., 2016).

### 2.7 Cytokine array and ELISA

HOrFs were co-cultured with EVs for 72 h as previously described. Twenty-four hours after the third time EVs had been added, the media was removed and replaced with serum-free media (without FGS and FBS). Forty-eight hours later, the conditioned serum free media was collected, spun at 2,000 rpm for 10 min at 4°C. The supernatant was then analyzed for 80 cytokines using the Human Cytokine Array C5 (RayBiotech) as per manufacturer instructions. Upon 30 min of blocking the arrays at RT, 1 mL of undiluted conditioned media was added to each well and incubated overnight at 4°C. After the first washing step, 1 mL of Biotinylated Antibody Cocktail was added to each well and incubated for 2 h at room temperature (RT). Upon second washing step, 2 mL of 1X HRP-Streptavidin was added to each well and incubated for 2 h at RT. Membranes were then washed a third time and then arranged for Chemiluminescence Detection using Detection Buffer using the ChemiDoc MP Imaging system (Bio-Rad). Background response was corrected using ImageJ Protein Array Analyzer (http://image.bio.methods.free.fr/dotblot.html). Dot values were measured using the same program. Normalization was performed per array manufacturer recommendations. Protein levels of CXCL5 and IL-8 in the cell culture supernatant were determined using enzyme linked immunosorbent assays (ELISA) assays (R and D Systems, Human IL-8 ELISA/CXCL8 and CXCL5/ENA-78 Quantikine ELISA Kits) as per manufacturer’s instructions.

### 2.8 Western blot

Protein was collected from EVs, cells or cell lysates through lysis with radioimmunoprecipitation assay (RIPA) buffer supplemented with 1:100 Protease Inhibitor Cocktail Set III and Phosphatase Inhibitor Cocktail 1 & 2 (Sigma-Aldrich). Protein was quantified using a Pierce BCA Protein Assay Kit (ThermoFisher). Ten μg of protein was separated using NuPAGE 4%–12% Bis-Tris Mini Protein Gels (ThermoFisher) and transferred to polyvinylidene difluoride membranes (Millipore). Membranes were blocked in 5% BSA, 1X TBS, and 0.1% Tween-20 at RT for 1 h. Membranes were then incubated overnight at 4°C with the appropriate primary antibody: 1:1000 diluted anti-Alix (sc-53540), anti-Hsp-70 (sc-24), anti-Flotillin-1 (sc-74566), anti-CD81 (sc-166029), anti-CD63 (sc-5275), and anti-GRP 94 (sc-393402) (all from Santa Cruz), and anti-TGFβ (#3711, Cell Signaling). After washing the membranes, they were subsequently incubated with peroxidase-conjugated 1:2000 Anti-Mouse (NXA931, GE Healthcare) or Anti-Rabbit (#7074, Cell Signaling). Detection was performed using Amersham ECL Western Blotting Detection Kit (GE Healthcare) and Chemidoc MP Imaging System (Bio-Rad).

### 2.9 Statistics

For flow cytometric data: a *p*-value of 0.05 or less was considered statistically significant (*p < 0.05, **p < 0.01, ***p < 0.001, ****p < 0.0001). Statistical analyses were performed using GraphPad Prism software (version 9.0, GraphPad Software, La Jolla, CA). All data are shown as mean ± S.E.M. For cell culture data, *n* equals the number of separate wells within the same experiment. For differential expression analysis of RNA sequencing data, corrected *p*-value <0.05 was considered statistically significant. For gene set enrichment, FDR q-value <0.25 along with nominal *p*-value <0.01 was considered enriched. For enrichment map visualisation, *p*-value cutoff: 0.05; FDR q-value cutoff: 0,1; overlap coefficient 0.5. Heatmap on specific CAF genes was done using z-scores calculated from FPKM values of differential expression analysis.

## 3 Results

### 3.1 EV characterization reveals high presence of small extracellular vesicles

**FIGURE 1:**
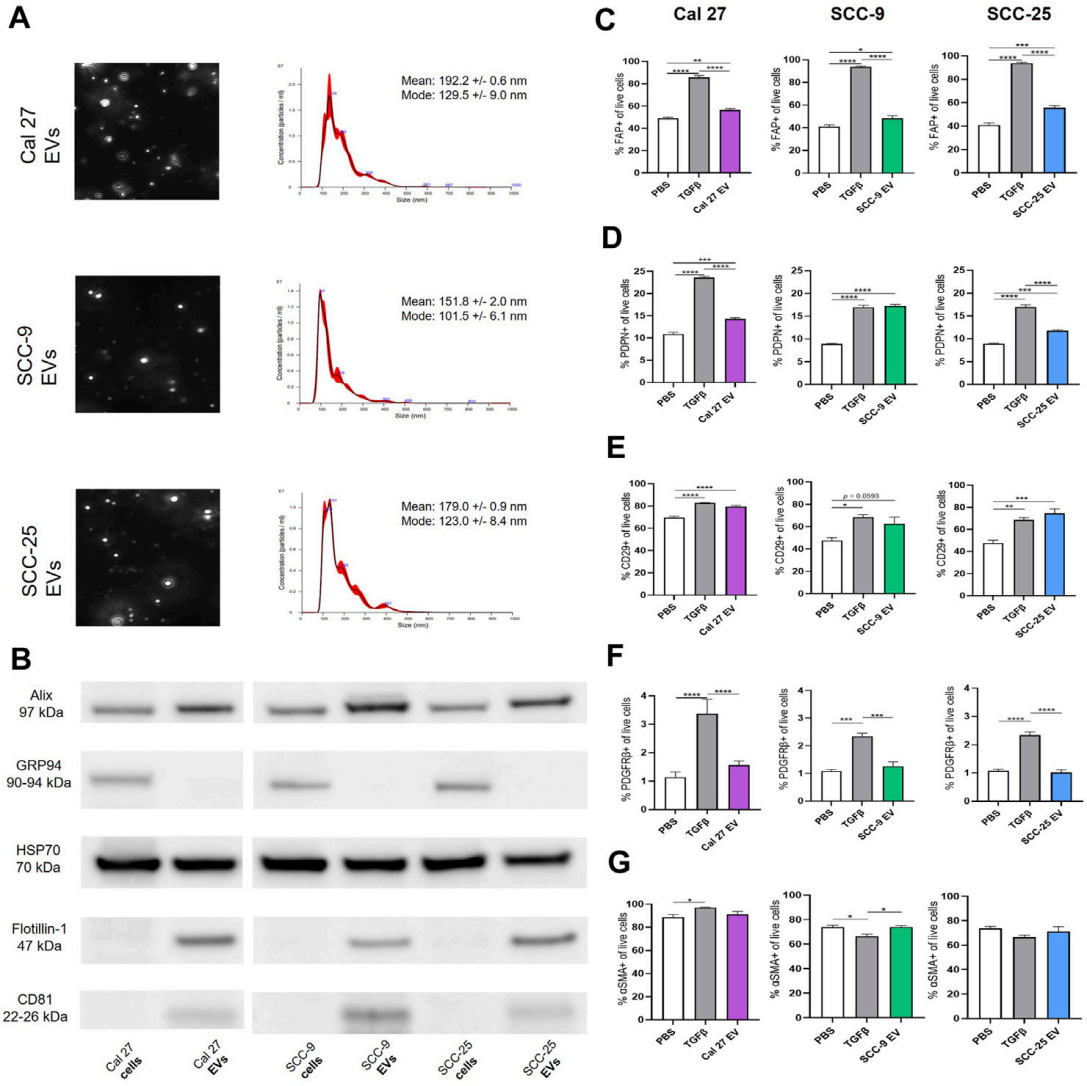
**(A)** Identification, quantification and characterization of representative EV particles from Cal 27, SCC-9 and SCC-25 cells according to NanoSight. Size distribution in nm, error bars indicate ± 1 SEM. **(B)** Protein from OSCC cell and EV protein tested for the vesicle markers Alix, HSP70, Flotillin-1, and CD81 together with GRP94 (negative marker for small EVs) with Western blot. **(C)**–**(G)** Flow cytometry analysis of percentage of fibroblasts positive for CAF markers upon co-culture with Cal 27, SCC-9, or SCC-25 derived EVs compared to PBS or TGFβ. **(C)** FAP, **(D)** PDPN, **(E)** CD29, **(F)** PDGFRβ, and **(G)** αSMA. *n* = 4. Statistics: Ordinary one-way ANOVA with Tukey’s multiple comparison test.

To understand the impact of oral tumor-derived EVs on surrounding fibroblasts, EVs from oral cancer cell lines Cal 27, SCC-9, and SCC-25 were first collected by repeated ultracentrifugation (Théry et al., 2018). The majority of EVs finally pelleted at 100,000 g consisted of exosomes ranging from 100 to 135 nm in size according to NanoSight analysis (Figure 1A). A peak was also seen around 180 nm, possibly partially containing microvesicles (Gould and Raposo, 2013). Western blot on isolated EVs as well as whole cell protein (the latter being source material) was performed confirming the presence of EV protein by EV markers Alix, HSP70, Flotillin-1, and CD81 (Figure 1B). GRP94 (also called HSP90B1) is a protein found in the endoplasmic reticulum. This protein is not present in EVs smaller than 200 nm and therefore was used as a control. GRP94 could not be detected in the EV protein (Figure 1B). Taken together, the collection method, size distribution, and protein content revealed the presence of EVs of the exosome type in the precipitate. This was used for further experiments.

### 3.2 EVs from oral cancer cells activate fibroblasts into cancer-associated fibroblasts (CAFs)

The currently accepted method for defining CAFs requires a combination of morphological features and biomarkers (Sahai et al., 2020). Various subtypes of CAFs have been identified expressing different CAF marker patterns (Costa et al., 2018; Givel et al., 2018; Galbo et al., 2021). We hypothesized that EVs from oral cancer cells could induce CAF activation and would give rise to CAFs with a unique expression profile.

To test this, human oral fibroblasts (HOrFs) were co-cultured with either PBS, TGFβ, or EVs isolated from oral cancer cell lines Cal 27, SCC-9, and SCC-25 cells for 72 h. Cell populations isolated from tumor tissue samples are defined as CAFs when they have an elongated spindle morphology, negative staining for non-mesenchymal biomarkers, and positive staining for mesenchymal biomarkers such as podoplanin (PDPN), CD29, alpha-smooth muscle actin (αSMA), fibroblast activation protein (FAP), and platelet-derived growth factor beta (PDGFβ) (Costa et al., 2018; Han et al., 2020). We analyzed CAFs by flow cytometry using antibodies for known CAF markers (FAP, PDPN, CD29, PDGFRβ, and αSMA). Flow cytometry analysis revealed a statistically significant increase in all three of the EV treatment groups compared to PBS for FAP, PDPN, and CD29 (Figures 1C–E). No difference was observed between the PBS and EV groups for PDGFRβ or αSMA (Figures 1F,G). TGFβ, a known activator of CAFs, was used as a positive control, resulting in an upregulation of FAP, PDPN, CD29, and PDGFRβ when compared to PBS (Figures 1C–F). The level of αSMA was generally high in all fibroblasts (co-cultured with EVs, TGFβ, or PBS) revealing no or week significances between groups (Figure 1G). This was possibly due to fibroblast activation linked to *in vitro* settings with cells cultured on stiff plastic surfaces, as previously experienced (Landry et al., 2019). See full gating procedures and representative dot blots in Supplementary Figure S1 and MFI data in Supplementary Figure S2. Western blot for TGFβ was performed on lysates obtained from the oral cancer cell lines as well as corresponding EVs to determine if TGFβ present in EVs could be a source of CAF activation (Supplementary Figure S2). Latent TGFβ was detected in both EVs and cell line lysates whereas activated TGFβ was only detected in EV protein from SCC-9 and SCC-25 cells, indicating a possible source of CAF activation.

### 3.3 RNA sequencing reveals different CAF signatures depending on activation pattern

**FIGURE 2:**
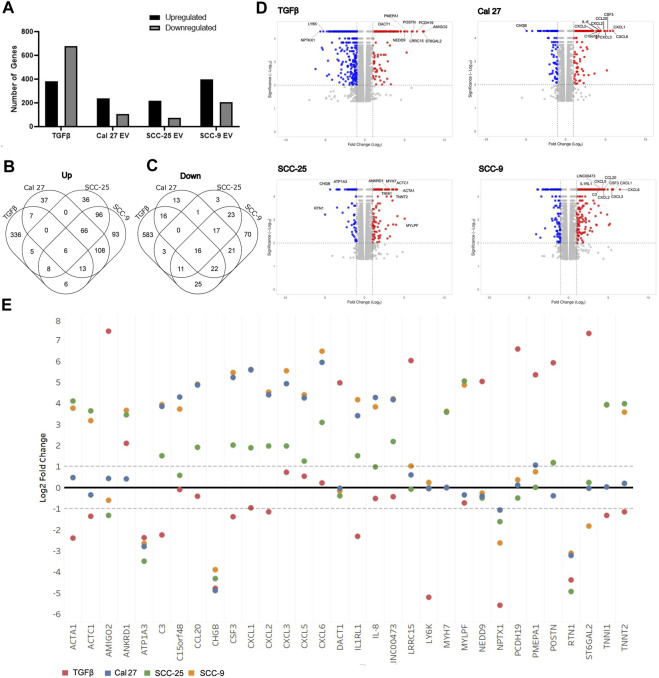
Overview of RNA sequencing of fibroblasts after treatment with OSCC cell line derived EVs or TGFβ. **(A)** Proportion of genes significantly up and downregulated compared to PBS control cells. Venn diagrams indicating overlap of upregulated **(B)** and downregulated **(C)** genes between treatment groups. **(D)** Volcano plots of differentially expressed genes in the four treatment types compared to PBS control. The top ten genes showing the highest combination of differential expression (taking into account fold change and statistical significance) are indicated. **(E)** Log2 fold change values for each treatment group for the top ten most highly differentially expressed genes in at least one sample (as shown in Figure 4D).

To determine if oral fibroblasts activated by TGFβ differed from those activated by oral tumor EVs, RNA from the activated fibroblasts was collected and RNA sequencing was performed. RNA was extracted from the activated fibroblasts using the same co-culture protocol as described above. Differential expression analysis was performed using Cufflinks confirming an upregulation in the RNA sequencing data of the CAF markers CD29, FAP, and PDPN in fibroblasts co-cultured with EVs seen with flow cytometry (except CD29 for fibroblasts co-cultured with EVs from SCC-25 cells) (Table 1). As expected, several additional known CAF markers (Öhlund et al., 2014; Gascard and Tlsty, 2016; Costa et al., 2018) were upregulated in all treatment groups (TGFβ and EVs) compared to PBS control, although overexpression was not necessarily observed across all samples for all markers, indicating different mechanisms of activation and/or function for resulting CAFs (Table 1). Similarly, expression of Caveolin-1 and Cavin-1 was not observed in CAFs activated by OSCC-derived EVs, as expected when fibroblasts are activated into CAFs (Table 1) (Öhlund et al., 2014; Gascard and Tlsty, 2016; Costa et al., 2018). In looking at the differences in the number of highly differentially expressed (DE) genes (q-value > 0.05 and log2 fold change >1) compared to control cells treated with PBS, we see marked differences in the profile of TGFβ-induced cells with a higher number of genes showing repression compared to the cells treated with EVs (Figure 2A), whereas the number of upregulated genes was relatively similar. The majority of differentially expressed genes in the cells with TGFβ treatment were unique to this treatment (88% of upregulated and 86% of repressed genes) (Figures 2B,C). Conversely, the majority of highly upregulated genes in the different cell line EV treatments showed an overlap in at least one other EV treatment, with 68%, 73%, and 75% of genes upregulated in Cal 27, SCC-9, and SCC-25 EV treatments, respectively, showing an overlap. Taken together, while TGFβ was detected in the EVs and may be a source of activation the differences between the TGFβ and EV treatments give evidence to distinct mechanisms of activation (Figures 2B,C). This is especially true for EVs from the Cal 27 cell line as TGFβ was not detected in the Cal 27 EVs and CAF activation was still observed and the expression profiles from all the EV treated groups clustered together and separated from the TGFβ treatment (Figures 2B,C).

**TABLE 1:** Differential expression analysis of CAF associated genes and whether genes where upregulated (*p* < 0.05) (gain) or downregulated (*p* < 0.05) (loss) or not (−) (see heading Affect), *p*-value, and relative fold change compared to PBS control (heading FC (log2)) in treatment groups with TGFβ or EVs compared to PBS. No upregulation in any treatment groups for the following genes: VIM, ACTA2, S100A4, NG2, FN1, MFAP5, ASPN, OGN, and SPARC. No downregulation in any treatment groups for the following gene: CD36.

Genes expected to show increased expression in CAFs												
CAF associated gene	HOrFs co-cultured with TGFβ			HOrFs co-cultured with cal 27 EVs			HOrFs co-cultured with SCC-9 EVs			HOrFs co-cultured with SCC-25 EVs		
	Affect	*p*-value	FC (log2)	Affect	*p*-value	FC (log2)	Affect	*p*-value	FC (log2)	Affect	*p*-value	FC (log2)
FAP	Gain	0.0003	2.035	Gain	0.0007	0.540	Gain	0.0003	0.479	Gain	0.0005	0.371
PDPN	Gain	0.0003	1.679	Gain	0.0007	1.909	Gain	0.0003	2.242	Gain	0.0005	1.426
ITGB1 (CD29)	Gain	0.0003	0.544	Gain	0.0182	0.264	Gain	0.0068	0.263	-	0.9247	0.014
PDGFRβ	Gain	0.0003	0.711	Loss	0.0177	−0.253	Loss	0.0003	−0.415	-	0.4460	−0.094
COL1A1	Gain	0.0003	0.873	Loss	0.0020	−0.304	-	0.1616	0.150	Gain	0.0005	0.695
COL1A2	Gain	0.0003	0.917	-	0.1824	−0.172	Gain	0.0003	0.452	Gain	0.0005	0.793
P4HA1	-	0.8021	−0.031	Gain	0.0007	0.342	Gain	0.0003	0.434	Gain	0.0005	0.402
P4HA2	Gain	0.0003	0.653	-	0.5734	0.099	Gain	0.0145	0.243	Gain	0.0005	0.349
P4HB	Gain	0.0066	0.257	-	0.3061	0.143	Gain	0.0247	0.218	-	0.2010	0.141
TNC	Gain	0.0003	0.403	Gain	0.0007	0.617	Gain	0.0003	0.364	-	0.4142	0.097
COL11A1	Gain	0.0003	0.634	-	0.2957	−0.189	Gain	0.0207	0.285	Gain	0.0005	0.504
ZEB1	Gain	0.0003	0.450	Gain	0.0166	0.263	-	0.9907	−0.002	-	0.1205	−0.169
MCT4	-	0.7737	0.034	Gain	0.0007	0.662	Gain	0.0003	0.645	Gain	0.0074	0.263
MMP1	Loss	0.0003	−1.375	Gain	0.0007	0.365	Loss	0.0379	−0.205	Loss	0.0005	−0.447
MMP2	Gain	0.0003	1.147	Gain	0.0464	0.226	Gain	0.0063	0.259	Gain	0.0409	0.210
MMP13	Gain	0.0069	1.970	-	1	−0.178	-	1	−1.092	-	1	0.024
MMP14	Gain	0.0003	0.921	-	0.9571	0.016	-	0.1798	−0.141	-	0.3165	−0.114
MMP19	Gain	0.0005	0.880	-	0.9960	−0.004	-	0.0620	−0.530	-	0.1941	−0.395
FOXF1	Loss	0.0003	−0.333	Gain	0.0036	0.293	-	0.4194	−0.095	Loss	0.0099	−0.281

Genes expected to show decreased expression in CAFs												
	Affect	*p*-value	FC (log2)	Affect	*p*-value	FC (log2)	Affect	*p*-value	FC (log2)	Affect	*p*-value	FC (log2)
Caveolin-1 (CAV1)	Loss	0.0003	−0.626	Loss	0.0007	−0.838	Loss	0.0003	−0.931	Loss	0.0005	−0.411
Cavin-1/PTRF	Gain	0.0003	0.454	Loss	0.0007	−0.545	Loss	0.0003	−0.785	Loss	0.0005	−0.551

Interestingly, when looking at the most highly differentially-expressed genes (Figures 2D,E), two of the OSCC cell lines EVs (SCC-9 and Cal 27) showed multiple cytokine/chemokine genes being upregulated in the CAFs after treatment with their EVs. Although SCC-25 did not have the same cohort of genes as highly differentially expressed, they still for the most part showed an obvious difference in the same genes, thus indicating that EV content plays a role in CAF expression. TGFβ treatment did not show this same trend (Figure 2E). Furthermore, when comparing the genes with the most highly differential expression (indicated in Figure 2D) for each treatment across all treatment types, the results of the EV-induced cells are much more similar to each other than the TGFβ-induced cells. Choosing four genes (CXCL5, IL-8, POSTN, and PMEPA1), qPCR validated the RNA sequencing data (Supplementary Figure S3). The increase in cytokine/chemokine expression and difference in expression from TGFβ treated fibroblasts indicates that these fibroblasts have likely polarized into an inflammatory-like fibroblast, analogous to the “iCAF” phenotype found in tumors (Biffi et al., 2019; Galbo et al., 2021). For full differential expression analysis, please see Supplementary Table S1.

### 3.4 CAFs activated through EVs show unique pro-inflammatory properties

Pathway analysis was performed to determine the impact of EV activation compared to activation by TGFβ, and to gain insight into the function of EV activated CAFs. Gene set enrichment analysis (GSEA) using Hallmarks gene sets was preformed revealing several enriched pathways (Supplementary Table S2), showing gene sets significantly enriched (FDR q-value < 0.25 and nominal *p*-value < 0.01)). Several of the significantly (*p*-value <0.01 and FDR >25%) enriched pathways in the CAFs activated by the three types of EVs were common in all three EV conditions and were distinct from the TGFβ group except for the EMT pathway, which was found to be enriched in all groups. Interestingly, pathways linked to inflammation were upregulated in all three cultures activated by the EVs (Supplementary Table S2, red box). All pathways involved in inflammation are illustrated in enrichment blots (Supplementary Figure S4) and enriched pathways are visualized in bar plots (Supplementary Figure S4).

**FIGURE 3:**
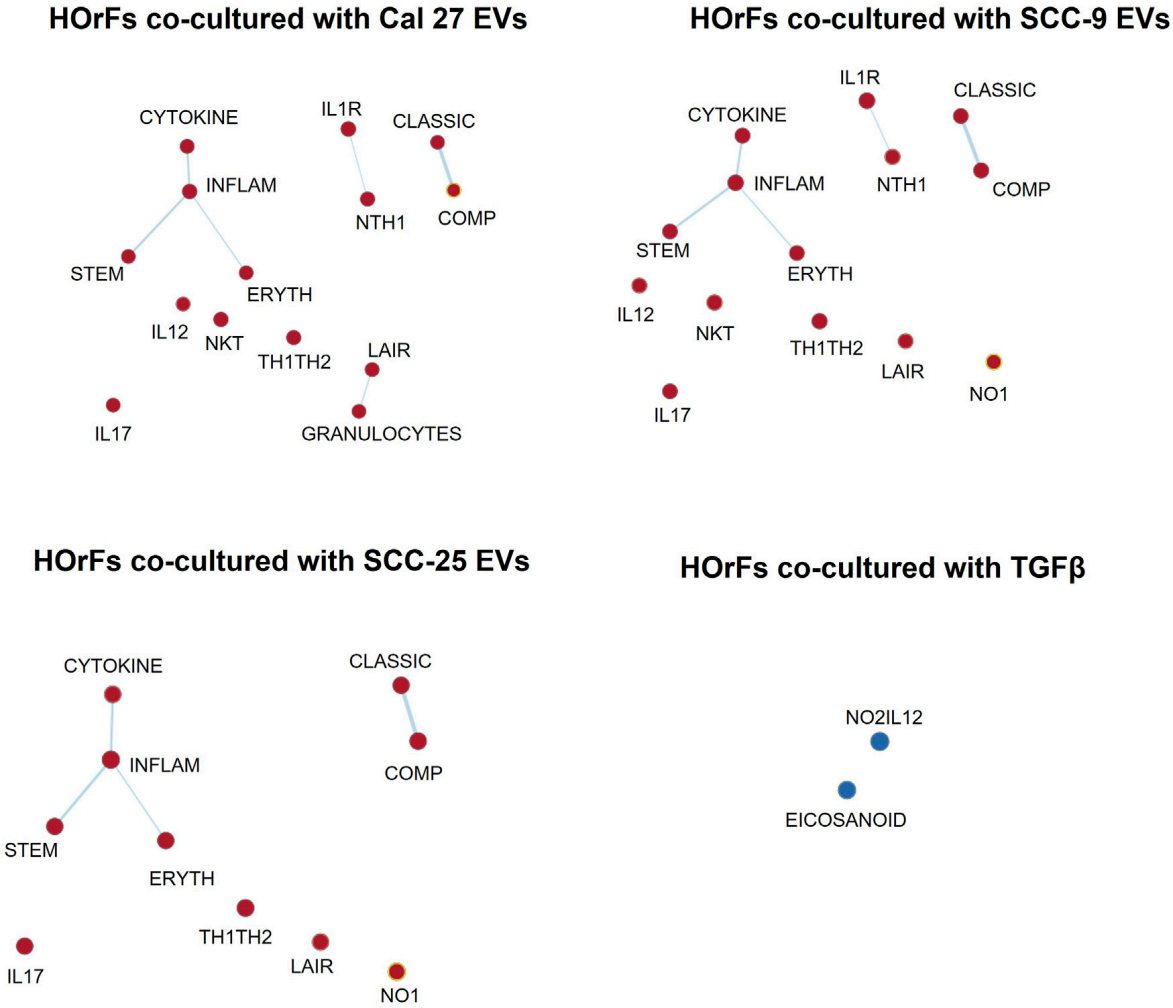
Enrichment map visualization. Commonalities between different enriched gene sets visualized through network analysis. Nodes are gene sets enriched (red circles) or depleted (blue circles). Lines link nodes with overlapping genes (bold line: high significance, thin line: moderate significance). Enrichment map visualization in GSEA was used together with Cytoscape 3.9.1. Enrichment Map Parameters: *p*-value cutoff: 0.05; FDR Q-value cutoff: 0.1; overlap coefficient: 0.5.

To determine possible interactions between different pathways, GSEA was performed using the more suitable Biocarta gene sets, since Hallmarks gene sets are created to avoid gene overlaps. An enrichment map was done visualizing enriched gene sets as a network, disclosing several overlapping genes between inflammatory pathways (Figure 3) (pathways described briefly in Supplementary Table S3). These maps emphasize the pro-inflammatory properties of CAFs activated through EVs from oral cancer cells. This was not seen in fibroblasts activated through TGFβ (Figure 3).

**FIGURE 4:**
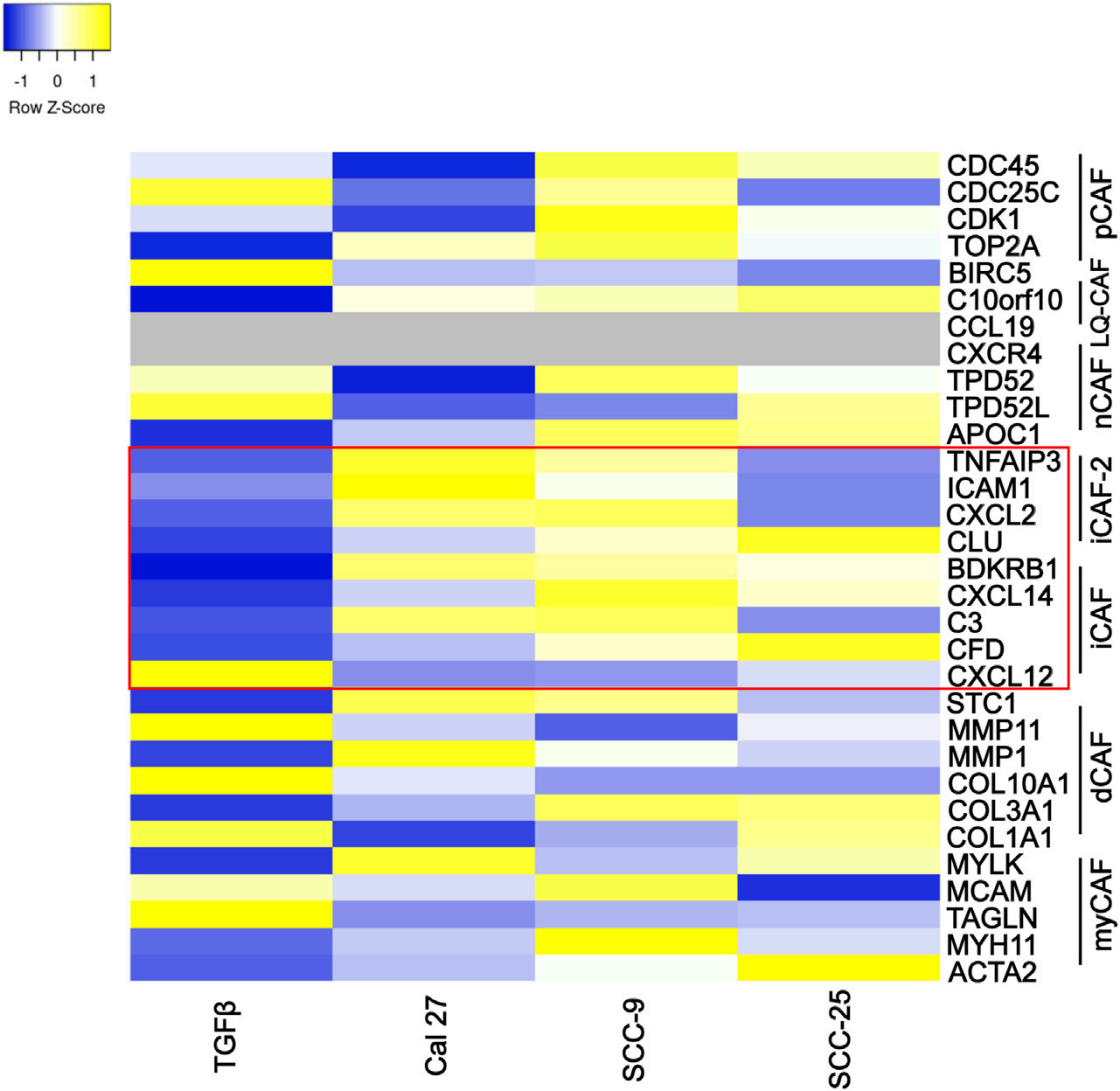
Specific marker genes related to pan-CAF subsets (right). Expression high (yellow), middle (white), low (blue), missing data (grey). Marker genes linked to pan-iCAF and pan-iCAF-2 marked with red rectangle. Z-scores calculated from FPKM values in differential expression analysis of fibroblasts co-cultured with TGFβ (TGFb), EVs from Cal 27 (CAL27), SCC-9 (SCC9) or SCC-25 (SCC25) cells, compared to fibroblasts co-cultured with PBS.

Several authors have described CAF subtypes in head and neck tumors (Costea et al., 2013; Patel et al., 2018; Galbo et al., 2021). Using several gene expression patterns, Galbo et al. defined six CAF subtypes (referred to as pan-CAF) (Galbo et al., 2021). To determine if the CAFs activated by oral cancer-associated EVs produced a specific CAF subtype (as defined by Galbo et al.) we analyzed the expression of gene sets that comprised each of the Galbo subtypes (Galbo et al., 2021). CAFs activated through co-culture with oral cancer EVs showed high presence of CAFs with pan-iCAF and pan-iCAF-2 signature (Figure 4). These two subsets have been described to have high expression of genes related to inflammation (Galbo et al., 2021). This was most evident in fibroblasts activated through co-culture with EVs from Cal 27 and SCC-9 cells (Figure 4), and not seen in fibroblasts activated through TGFβ. CAFs activated with SCC-25-derived EVs where more similar to the other EV groups than to TGFβ activated CAFs but appear to have a distinct expression pattern. This is not surprising as the EV miRNA content of EVs from SCC-25 cells is also distinct from the EV content from SCC-9 and Cal 27 cells (see Supplementary Figure S5) (Dickman et al., 2017).

Taken together, pathway analysis, enrichment map visualization, and subtype analysis all suggest that oral CAFs activated through EVs from oral cancer cells show pro-inflammatory properties unique from CAFs activated through TGFβ.

### 3.5 CAFs activated through EVs secrete the pro-inflammatory cytokines IL-8 and CXCL5

**FIGURE 5:**
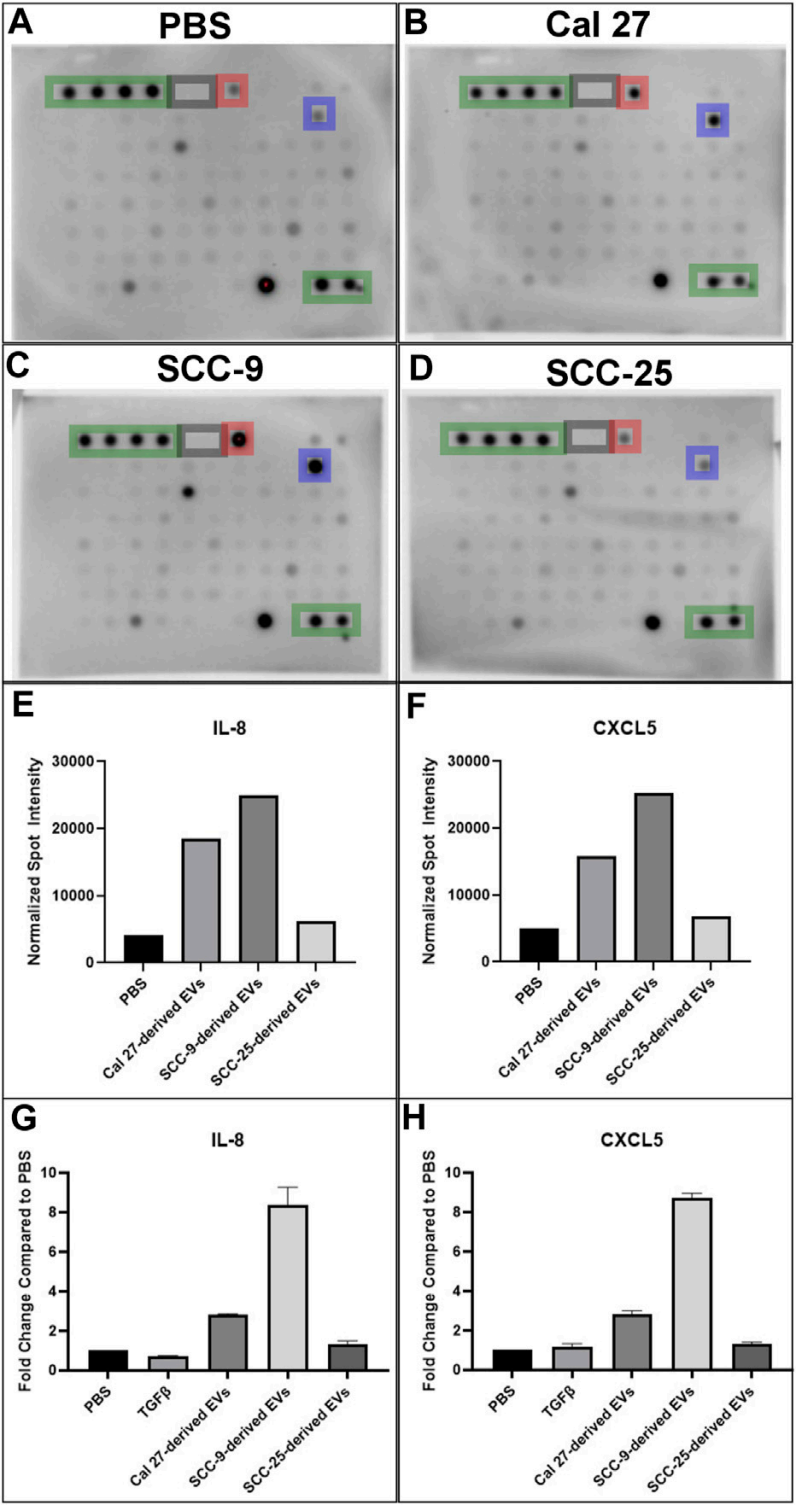
Cytokine antibody array profiles **(A–F)** and ELISA **(G–H)** of conditioned media from HOrF cell culture media upon 72 h of co-culture. **(A)** Co-culture with PBS. **(B)** Co-culture with EVs derived from Cal 27 cells. **(C)** Co-culture with EVs derived from SCC-9 cells. **(D)** Co-culture with EVs derived from SCC-25 cells. Positive control spots are outlined in green and negative in gray. Spots corresponding to IL-8 are highlighted in blue and CXCL5 in red. **(E)** Bar graphs indicating increased protein expression of IL-8 and **(F)** CXCL5. ELISA on **(G)** IL-8 and **(H)** CXCL5.

Cells with pro-inflammatory properties are known to secrete a distinct pattern of cytokines that promote inflammation (Kopf et al., 2010; Deckers et al., 2023). To further understand the role of EV-activated CAFs, conditioned media from CAFs activated by oral cancer cell EVs was collected for cytokine analysis using a screening membrane-based antibody array analyzing 80 different cytokines. Out of a total of 80 cytokines profiled on the array, 76 were detected in at least one of the samples (Supplementary Table S4). The majority of cytokines were detected at similar levels compared to the PBS control; however, 16 were differentially expressed in conditioned media from at least 1 cell line cultured with EVs as compared to the PBS control. The majority of proteins were over-expressed (14/16) and only two proteins were under-expressed compared to the PBS control in one of the cell lines (Cal 27) (Table 2). The most highly differentially expressed proteins in the conditioned media from cells cultured with EVs were IL-8 (CXCL8) and CXCL5 (ENA-78). These two factors had increased relative expression in two out of the three EV treated cell lines (Cal 27 and SCC-9) (Figures 5A–F). To validate the data, an ELISA was performed on the conditioned media from CAFs activated by oral cancer cell EVs or TGFβ, displaying CAFs activated by Cal 27 or SCC-9-derived EVs to secrete upregulated levels of IL-8 and CXCL5 compared to PBS treated fibroblasts (Figures 5G, H). Correspondingly, IL-8 and CXCL5 were significantly upregulated in CAFs activated through oral cancer cell EVs (from Cal 27, SCC-9, and SCC-25 cells) according to differential expression analysis of RNA sequencing data (Supplementary Table S1).

**TABLE 2:** Relative fold change values for differentially expressed proteins on cytokine antibody array.

Upregulated proteins	Relative fold change compared to PBS control		
	Cal27	SCC-9	SCC-25
IL-8	4.480	6.044	1.502
CXCL5	3.136	4.997	1.342
GRO-alpha	1.272	5.170	1.668
MCP-1	0.813	2.047	1.086
GRO	1.384	2.824	0.903
IL-1alpha	2.235	1.612	1.423
IL-10	2.390	1.850	1.233
TNF-beta	1.001	0.865	7.263
TNF-alpha	0.881	0.865	4.272
EGF	1.178	0.958	3.486
IGF-1	1.850	1.026	3.292
TGF-beta 1	1.042	0.989	3.288
Eotaxin-3	1.491	0.857	3.229
PIGF	1.155	1.011	2.067

Downregulated Proteins	Relative Fold Change Compared to PBS Control		
	Cal27	SCC-9	SCC-25
HGF	0.37	0.86	0.71
Osteoprotegerin	0.48	1.11	0.96

## 4 Discussion

Several subtypes of CAFs have been identified (Costea et al., 2013; Costa et al., 2018; Givel et al., 2018; Patel et al., 2018; Galbo et al., 2021). However, their specific function and how they are generated is not fully understood.

TGFβ family ligands constitutes well-established activating signals for myofibroblast-like fibroblasts (Sahai et al., 2020). In this study we demonstrate that CAFs activated through oral cancer-derived EVs induce an iCAF phenotype. GSEA of CAFs activated by oral cancer-derived EVs revealed upregulation of several pathways linked to inflammation including TNFα, IL6_JAK_STAT3 pathway, and IL2_STAT5 signaling pathway (Supplementary Table S2, red box). The NF-κB family of transcription factors are widely described as central for inducing genes regulating an inflammatory response, both through the innate and adaptive immune system (Liu et al., 2017). The IL-6 pathway stimulates acute phase responses and immune reactions. Elevated expression of IL-6 leads to activation of JAK-STAT signaling, a cornerstone to cancer progression in particular linked to STAT3 activation, both through tumor growth and metastasis as well as modulation of immune surveillance (Brooks and Putoczki, 2020). CAFs activated by oral cancer-derived EVs revealed upregulation of the pro-inflammatory gene IL-6, not seen in CAFs activated by TGFβ (Supplementary Table S1) and iCAFs are known to secrete IL-6 (Biffi et al., 2019).

CAFs activated by EVs also showed upregulated genes in response to low oxygen levels (hypoxia pathway), not seen in CAFs activated by TGFβ. Hypoxia related genes NF-κB, HIF1α, HK2, and PFKL (Ikeda et al., 2020; Baqlouq et al., 2021) were upregulated in CAFs activated by oral cancer-derived EVs (Supplementary Table S1). Only NF-κB was upregulated in CAFs activated by TGFβ (Supplementary Table S1). Tumors with high proportion of hypoxic cells have worse therapeutic outcome linked both to surgery and radiation, and patients with hypoxic tumors have increased risk for metastases and worse overall survival (Wadsworth et al., 2022). Hypoxia has previously been linked to IL-1 induced iCAFs (Biffi et al., 2019; Schwörer et al., 2023).

The Interferone_gamma_response and the complement pathways were also upregulated in CAFs activated by EVs. In the TME, IFN-γ and the complement system consistently orchestrate both pro-tumorigenic and antitumor immunity overall (Jorgovanovic et al., 2020; Revel et al., 2020). The IFN-γ pathway has been shown to be enriched in iCAFs, with known roles in cancer progression and disease pathophysiology (Öhlund et al., 2014).

GSEA revealed several overlapping genes between inflammatory pathways, strengthening the suggested pro-inflammatory profile of CAFs activated through oral cancer cell EVs. In line with these results, cytokine analysis of the CAF secreted proteins revealed 14 upregulated cytokines in EV activated CAF supernatant compared to supernatant from fibroblasts that received only PBS, with IL-8 and CXCL5 being the cytokines most upregulated (Figure 5). IL-8 and CXCL5 were also among the most highly over-expressed genes in the EV activated CAFs compared to the PBS treatment (Supplementary Table S1). The possible importance of IL-8 can not be underestimated. IL-8 (CXCL8) is a pro-inflammatory chemokine that induces neutrophil degranulation and is vital during systemic inflammation (Bernhard et al., 2021). In cancer, it enhances tumor cell growth and metastasis through epithelial to mesenchymal transition (Fernando et al., 2011), formation of neutrophil extracellular traps (Alfaro et al., 2016), angiogenesis and infiltration of inflammatory cells that suppress antitumor CD3+/CD8+ T cell functions (David et al., 2016). Cancer immunology has to a large extent been focused on T cells and CD3+/CD8+ T cell response is linked to cancer survival (Fridman et al., 2012; Jimenez et al., 2021). The role of neutrophils in cancer is heterogenous. However, most studies support a pro-tumoral role for these abundant myeloid cells (Millrud et al., 2017; Hedrick and Malanchi, 2022). The effects of IL-8 linked to neutrophils and CD3+/CD8+ cells are thus of great importance in tumor biology. Additionally, high plasma levels of IL-8 have been associated with decreased efficiency of PD-L1 blockade in metastatic urothelial and renal cell carcinoma (Yuen et al., 2020). The small inflammatory cytokine CXCL5 activates neutrophils and mediates neutrophil infiltration. CXCL5 directly induces cancer cell proliferation and invasion and has been reported to be over-expressed in several cancer forms, including squamous cell cancer (Miyazaki et al., 2006; Wente et al., 2006; Park et al., 2007; Wong et al., 2007; Kuo et al., 2011). Altogether, this suggests CAFs activated by tumor EVs can shape the immunosuppressive microenvironment.

CAFs have been shown to have both pro-tumorigenic as well as anti-tumorigenic effects. The presence of CAF subtypes with different marker patterns at least partially explains this. There are several studies describing CAF subtypes in cancer. In the areas of breast and ovarian cancer, CAF subtypes have been defined based on multicolor flow cytometry yielding the four subtypes depending on how they stained for the fibroblast markers FAP, CD29, αSMA, S100-A4, PDGFRβ, and CAV1, accumulating differently in different tumors (Costa et al., 2018; Givel et al., 2018). The oral fibroblasts we describe in this manuscript, that were activated into CAFs through oral cancer cell-derived EVs, most likely constitute the CAF-S1 subtype, positive for both CD29 and FAP, while lacking expression of CAV1 (Table 1). CAF-S1 is described as having immunosuppressive functions by attracting CD25+FOXP3+ T cells. This effect was shown to be dependent on CXCL12β isoform mediated by IL-6 (Givel et al., 2018). CAFs activated by OSCC-derived EVs revealed in line with this upregulated IL-6 (Supplementary Table S1). However, CXCL12 showed no upregulation, possibly due to inability at RNA sequencing and cytokine profiling to distinguish the β-isoform from the α-isoform (Supplementary Table S1–S4). Further functional studies investigating what effects oral CAFs activated through oral cancer-derived EVs have on CD25+FOXP3+ T cells would hence be highly interesting. As suggested by Costa et al., targeting CAF subtypes might be a promising therapeutic approach as a complement to established treatment management.

CAF subtypes have also been described in oral cancer (Costea et al., 2013; Patel et al., 2018; Galbo et al., 2021). Using RNA sequencing, Galbo et al. (Galbo et al., 2021) compared CAFs across multiple cancer types, including head and neck squamous cell carcinoma, to identify CAF subtypes characterized by different cell-surface pattern and with diverse critical molecular pathways activated. According to our data, CAFs activated through oral cancer-derived EVs showed similar gene expression patterns as previously described pan-iCAFs and pan-iCAF-2 with high expression of genes related to inflammation (Figure 4). This was not seen in CAFs activated through TGFβ, displaying more similarities with pan-pCAFs and pan-dCAFs. Pan-iCAFs and pan-iCAF-2 have low expression of collagens but high expression of pro-inflammatory molecules and the transcription factors NR1H3 and the NFkB subunit RELB, both promoting inflammatory transcriptional programs (Galbo et al., 2021). These results suggests that pan-iCAFs and pan-iCAFs-2 are potentially associated with a molecular microenvironment with enhanced immune cell activation. CAFs activated by SCC-25-derived EVs did not display pan-iCAFs and pan-iCAFs-2 as distinct as CAFs activated through EVs from Cal 27 or SCC-9 cells (Figure 4). This suggest the cell specific EV content may be crucial for CAF subtype differentiation. Several components in EV cargo have been shown to affect stromal cells including miRNAs, TGFβ, cells surface proteins, and other proteins, as well as mRNAs and lncRNAs (Dai et al., 2018; Shoucair et al., 2020; Zhang et al., 2022). The EV content that drives CAF activation is still under investigation, however it is tempting to speculate miRNA cargo to be of importance (Supplementary Figure S4). Intercellular differences were seen regarding EV miRNA content that could explain the small differences seen in activation of inflammatory CAFs. Future studies looking at key players in EV cargo activating inflammatory CAFs and at tissue samples for the various CAF subtypes that are actually present in patients and in what frequency would be crucial in understanding the role for specific CAF subtypes.

## Data Availability

The datasets presented in this study can be found in online repositories. The names of the repository/repositories and accession number(s) can be found below: https://www.ncbi.nlm.nih.gov/geo/query/acc.cgi?acc=GSE233618.
