# Supplementary material for: Extracellular vesicles promote activation of pro-inflammatory cancer-associated fibroblasts in oral cancer
Source: Front Cell Dev Biol. 2023 Sep 7;11:1240159. doi: 10.3389/fcell.2023.1240159 (PMC10513103; doi:10.3389/fcell.2023.1240159)
Supplement: Supplementary file 2 [file DataSheet1.pdf]

Supplementary Material

Extracellular vesicles promote activation of pro-inflammatory cancer-associated fibroblasts in oral cancer

Julia Arebro[*], Rebecca Towle, Che-Min Lee, Kevin Bennewith, Cathie Garnis

*** Correspondence:** Corresponding Author: julia.arebro@regionstockholm.se

1 Supplementary Figures and Tables

1.1 Supplementary Figures

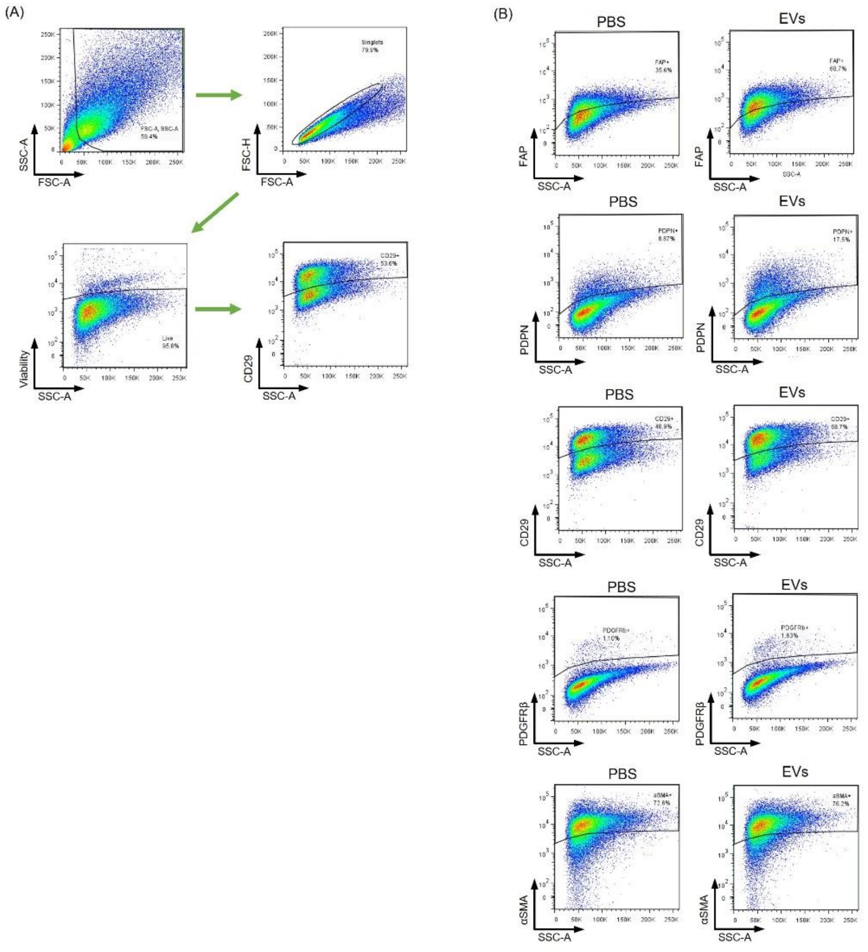

Supplementary Figure S1. (A) Representative gating procedures and (B) representative dot plots of fibroblasts positive for CAF markers upon co-culture with Cal 27, SCC-9, or SCC-25-derived EVs compared to PBS.

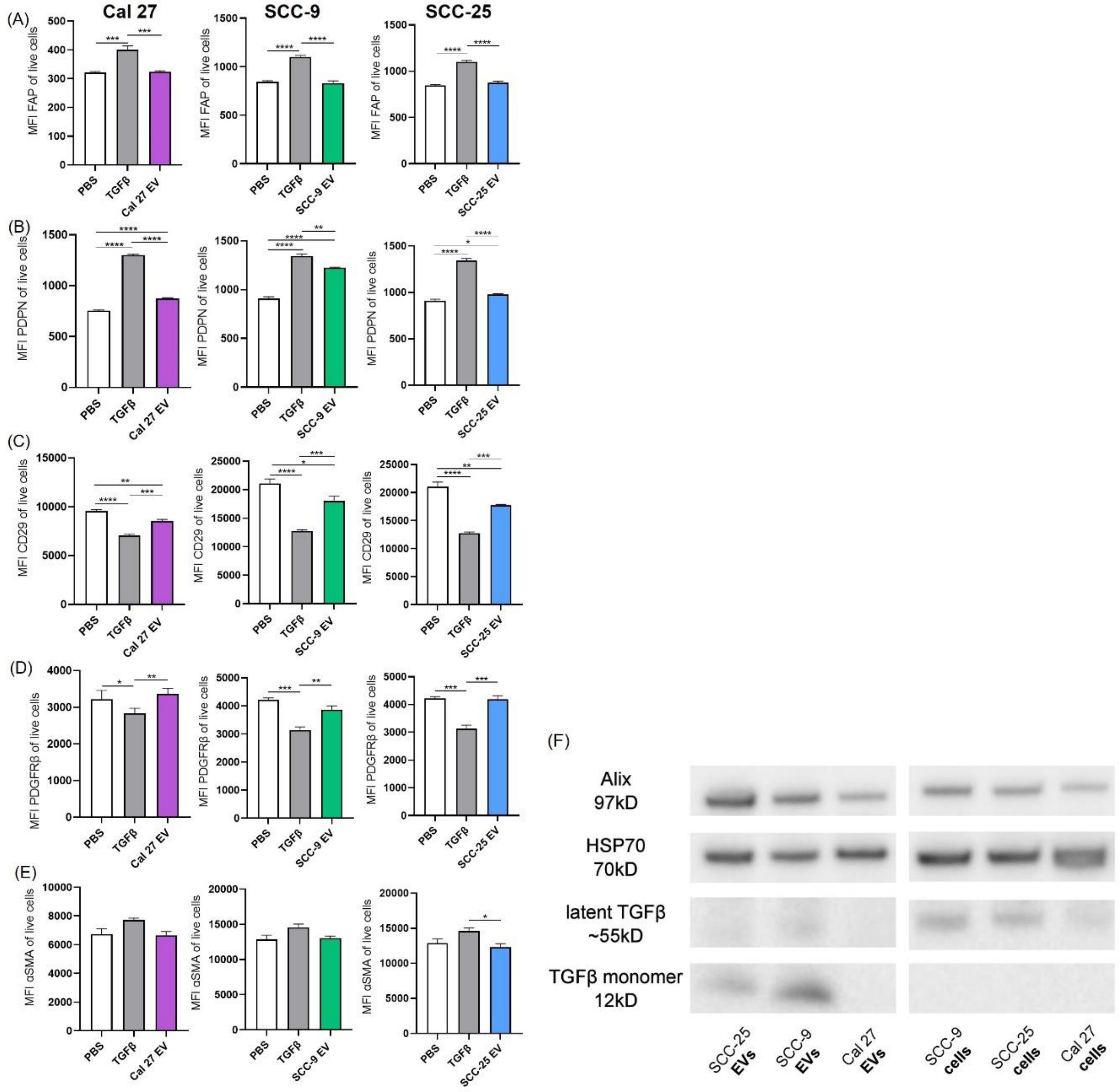

Supplementary Figure S2. Flow cytometry analysis of MFI of CAF markers in fibroblasts co-cultured with Cal 27, SCC-9, or SCC-25-derived EVs compared to PBS or TGFβ. (A) FAP, (B) PDPN, (C) CD29, (D) PDGFRβ, and (E) αSMA. *n* = 4. Statistics: Ordinary one-way ANOVA with

Tukey's multiple comparison test. (F) Western blot on TGFβ performed on oral cancer cell lysates and EVs. Alix and HSP70 used as a positive control.

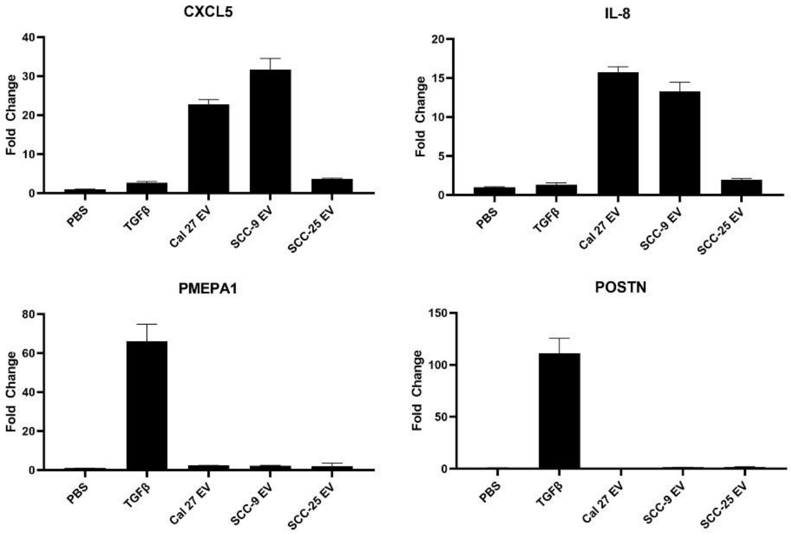

Supplementary Figure S3. qPCR results of CXCL5, CXCL8, PMEPA1, and POSTN in HOrF cells treated with either PBS, TGFβ, Cal 27-derived, SCC-9-derived, or SCC-25-derived EVs. GAPDH was used as the endogenous control. Fold change calculated compared to PBS treated samples.

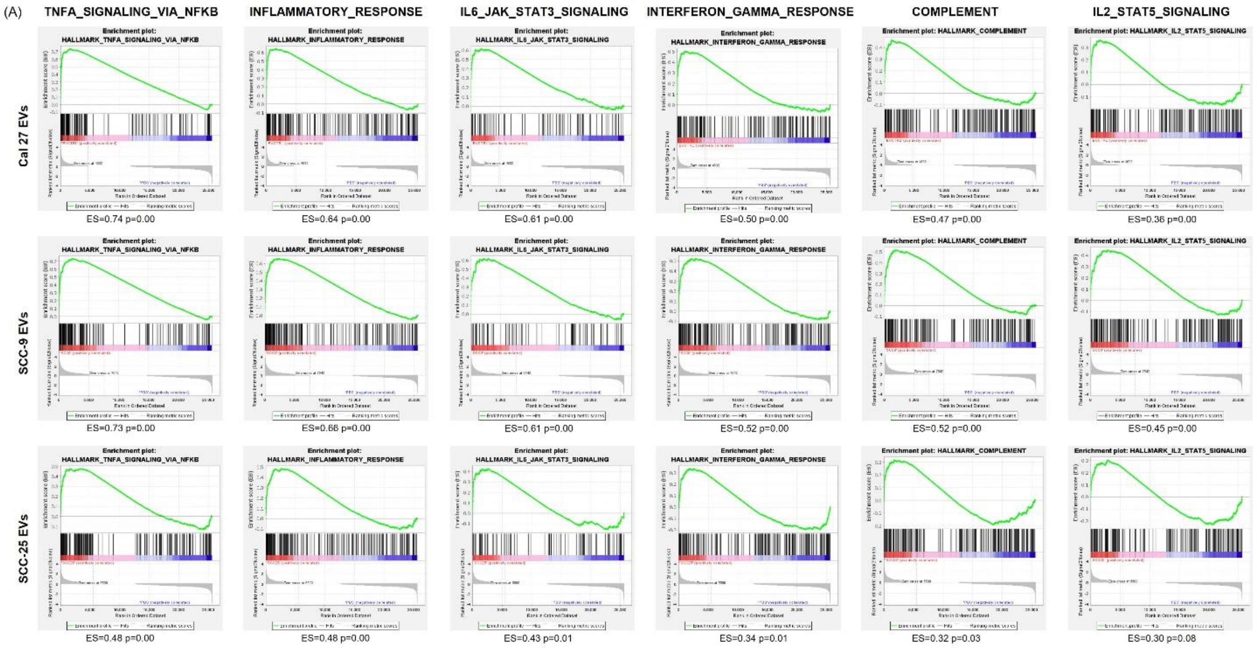
(A)
TNFA_SIGNALING_VIA_NFKB
INFLAMMATORY_RESPONSE
IL6_JAK_STAT3_SIGNALING
INTERFERON_GAMMA_RESPONSE
COMPLEMENT
IL2_STAT5_SIGNALING
Cal 27 EVs
SCC-9 EVs
SCC-25 EVs
ES=0.74 p=0.00
ES=0.64 p=0.00
ES=0.61 p=0.00
ES=0.50 p=0.00
ES=0.47 p=0.00
ES=0.36 p=0.00
ES=0.73 p=0.00
ES=0.66 p=0.00
ES=0.61 p=0.00
ES=0.52 p=0.00
ES=0.52 p=0.00
ES=0.45 p=0.00
ES=0.48 p=0.00
ES=0.48 p=0.00
ES=0.43 p=0.01
ES=0.34 p=0.01
ES=0.32 p=0.03
ES=0.30 p=0.08

(B)

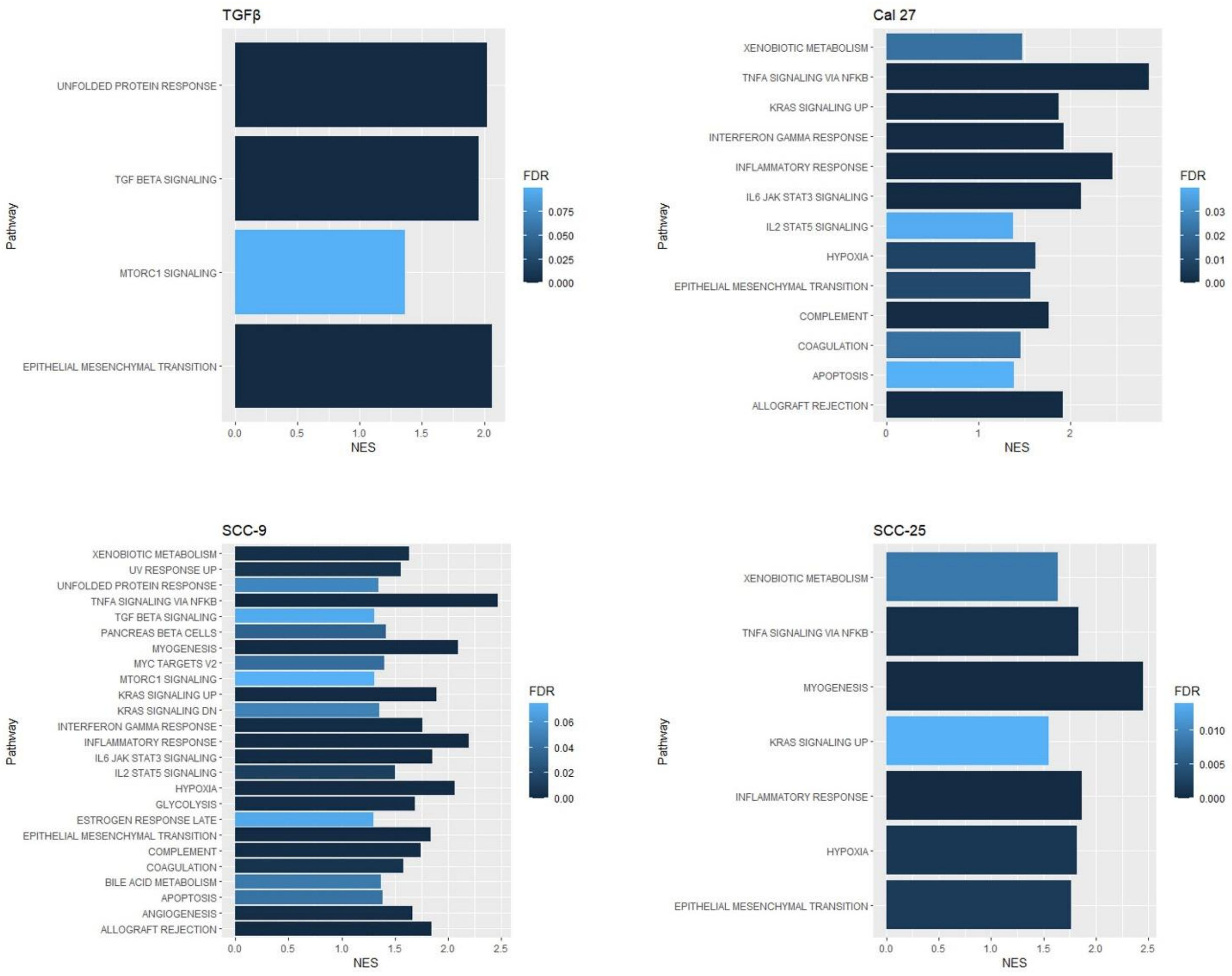

Supplementary Figure S4. (A) Enrichment blots of inflammatory pathways (Hallmark gene sets) in fibroblasts co-cultured with EVs versus PBS with enrichment score (ES) and nominal p-values (p). (B) Bar plots of enriched pathways (Supplementary Table S2) with normalized enrichment score (NES) and FDR.

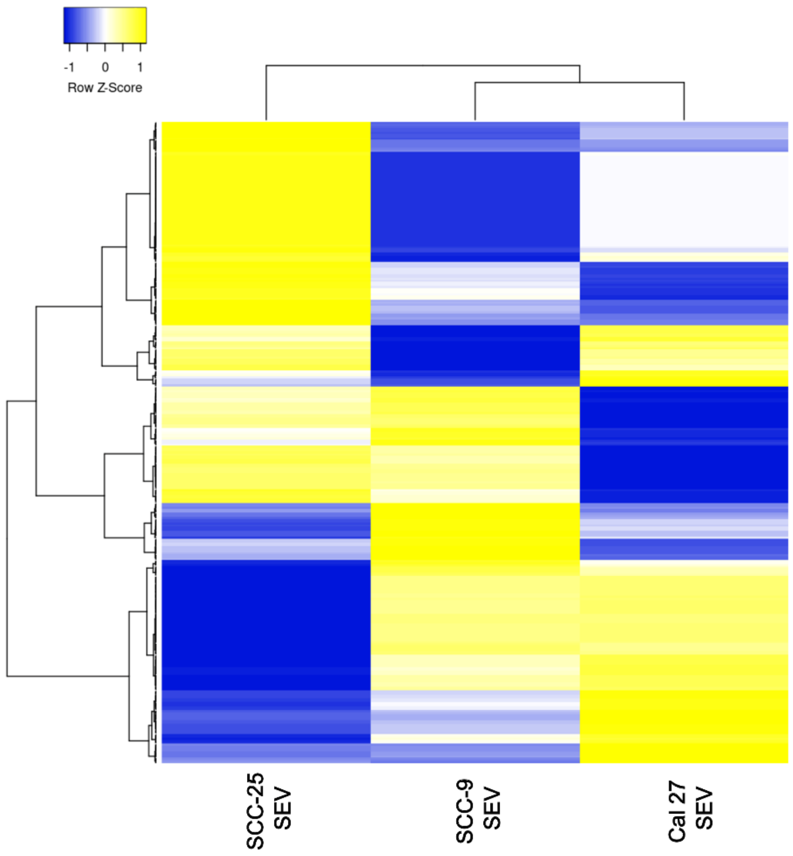

Supplementary Figure S5. Unsupervised hierarchical clustering analysis of miRNAs profiled from the small EVs (SEV) collected from Cal 27, SCC-9 and SCC-25 cells. Data from Dickman et al 2017.

1.2 Supplementary Tables

TGFβ	Cal 27	SCC-9	SCC-25
UNFOLDED_PROTEIN_RESPONSE	TNFA_SIGNALING_VIA_NFKB	TNFA_SIGNALING_VIA_NFKB	TNFA_SIGNALING_VIA_NFKB
TGF_BETA_SIGNALING	INFLAMMATORY RESPONSE	INFLAMMATORY RESPONSE	INFLAMMATORY RESPONSE
MTORC1_SIGNALING	IL6_JAK_STAT3_SIGNALING	IL6_JAK_STAT3_SIGNALING	IL6_JAK_STAT3_SIGNALING
	INTERFERONE_GAMMA_RESPONSE	INTERFERONE_GAMMA_RESPONSE	
	COMPLEMENT	COMPLEMENT	
	IL2_STAT5_SIGNALING	IL2_STAT5_SIGNALING	
	KRAS_SIGNALING_UP	KRAS_SIGNALING_UP	KRAS_SIGNALING_UP
	HYPOXIA	HYPOXIA	HYPOXIA
	ALLOGRAFT_REJECTION	ALLOGRAFT_REJECTION	ALLOGRAFT_REJECTION
	XENOBIOTIC_METABOLISM	XENOBIOTIC_METABOLISM	XENOBIOTIC_METABOLISM
	MYOGENESIS	MYOGENESIS	MYOGENESIS
EPITHELIAL_MESENCHYMAL_TRANSITION	EPITHELIAL_MESENCHYMAL_TRANSITION	EPITHELIAL_MESENCHYMAL_TRANSITION	EPITHELIAL_MESENCHYMAL_TRANSITION
	APOPTOSIS	ANGIOGENESIS	
		COAGULATION	KRAS_SIGNALING_DN
		UV_RESPONSE_UP	GLYCOLYSIS
		GLYCOLYSIS	

Supplementary Table S2. Gene sets significantly enriched (FDR q-value <0.25 and nominal p-value <0.01) in treatment group vs PBS group according to GSEA and Hallmarks gene sets. Pathways linked to inflammation marked with red box.

Pathway	Description
CYTOKINE_PATHWAY	Cytokine Network for communication between different immune cells
INFLAM_PATHWAY	Cytokines and Inflammatory Response
STEM_PATHWAY	Regulation of hematopoiesis by cytokines
ERYTH_PATHWAY	Erythrocyte Differentiation Pathway
IL1R_PATHWAY	Signal transduction through IL1R, the receptor to the pro-inflammatory cytokine IL-1
NTHI_PATHWAY	NFkB activation by Nontypeable Hemophilus influenzae
LAIR_PATHWAY	Cells and Molecules involved in local acute inflammatory response
GRANULOCYTES_PATHWAY	Adhesion and Diapedesis of Granulocytes
CLASSIC_PATHWAY	Classical Complement Pathway, stimulates phagocytosis of foreign cells and an inflammatory response
COMP_PATHWAY	Complement pathway, consisting of a series of over thirty proteins in plasma that are part of the immune response

Supplementary Table S3. Brief description of pathways (Biocarta gene set) with connections visualized through network analysis in CAFs activated through co-culture with oral cancer-derived EVs.

2 Supplementary Materials and methods

2.1 qPCR

RNA was extracted using the TRIzol protocol as per manufacturer's instructions (Thermofisher) with residual DNA removed using the DNA-Free DNA removal kit (Thermofisher) according to manufacturer's instructions. RNA was converted to cDNA using the Applied Biosystems High Capacity Reverse Transcription kit (Thermofisher). Taqman gene expression assays for CXCL8 (Hs00174103_m1), CXCL5 (Hs01099660_g1), POSTN (Hs01566750_m1) and PMEPA1 (Hs00375306_m1) were used, with GAPDH (Hs02758991_g1) as the endogenous control. qPCR reactions were performed using Taqman universal master mix II, no UNG (Thermofisher) on Applied Biosystems ViiA7 machine (Thermofisher). The delta-delta-Ct method was used to determine fold change compared to a control.
